# Proximal myopathy: causes and associated conditions

**DOI:** 10.15190/d.2022.19

**Published:** 2022-12-31

**Authors:** Amina Rao, Iqra Nawaz, Fawad Mueen Arbi, Rizwan Ishtiaq

**Affiliations:** ^1^Quaid-e-Azam Medical College, Bahawalpur, Pakistan; ^2^St. Francis Hospital, Hartford, CT, USA

**Keywords:** Muscular diseases, myotoxicity, neuromuscular diseases, myopathies, structural, congenital, musculoskeletal diseases.

## Abstract

Proximal myopathy presents as generalized muscle weakness commonly involving the muscles of upper and/or lower limbs. Toxins, long-term use of statins, corticosteroids, alcohol, SGLT2 inhibitors, COVID-19 vaccination, and antimalarials have been attributed to its development. In endocrine and metabolic disorders, adrenal dysfunction including both overproduction and insufficiency of the adrenal gland hormones has been reported to cause myopathy. Moreover, parathyroid and thyroid disorders along with pituitary gland disorders can also directly or indirectly contribute to this condition. In idiopathic inflammatory myopathies including polymyositis, dermatomyositis, inclusion body myositis (IBM), and Systemic Lupus Erythematosus (SLE), Sjögren’s Syndrome, and overlap syndromes, moderate to severe muscle weakness has been observed. IBM has been reported to be the most prevalent acquired myopathy above the age of 50. Hereditary or congenital myopathies include limb girdle muscular dystrophies, facioscapulohumeral muscular dystrophy, Duchenne and Becker muscular dystrophy, and proximal myotonic myopathy. In addition to these, glycogen storage diseases such as the McArdle disease can also cause fast exhaustion, myalgia, and cramping in working muscles. It is pertinent to mention here that a class of hereditary metabolic myopathies, referred to as "lipid deposition myopathy" causes lipids to accumulate in skeletal muscle fibers, leading to lesions and degeneration. Among viral causes, HIV, dengue virus, influenza virus, hepatitis B virus, hepatitis C virus, SARS-CoV2 are also associated with muscle weakness. Sarcoidosis, an inflammatory disease, can also manifest as muscle weakness and myalgia. Owing to this complicated pathophysiology of proximal myopathy, this review aims to summarize the existing literature on conditions associated with this phenomenon and other recent developments that have been made regarding events leading to development of generalized muscle weakness. To the authors’ knowledge this is the first narrative review that discusses causes and conditions associated with proximal myopathy in thorough detail.

## 1. Introduction

As the name implies, the term myopathy literally refers to muscle disease. The pattern of weakness observed in this phenomenon commonly involves muscles of upper and/or lower limb and less commonly muscles of neck, face, distal limb, eye, pharynx, respiratory system, and heart. Patients present with generalized weakness and muscle pain which disturb their everyday lives^1^. In myopathies, the most common pattern is symmetric weakness, which most commonly affects the proximal muscles of limbs^2^. The more prevalent kind of myopathy is inflammatory and endocrine, which often affects middle-aged women as compared to males. Inflammatory myopathies have been reported to have an incidence rate of 1.16 to 19 per million people per year and a prevalence of 2.4 to 33.8 per 100,000 people^3^. Duchenne's and Becker's muscular dystrophies are the most prevalent forms of hereditary proximal myopathies, with prevalence rates ranging from 19.8 to 25.1 per 100,000 person/years^4,5^. Over the course of last few years, with more developments in scientific knowledge, the clinical management of proximal myopathy has changed accordingly. Through this review we aim to organize and present these new developments pertaining to the causes and conditions associated with this phenomenon in the form of an elaborate summary as the knowledge of all known factors contributing to the development of proximal myopathy is necessary for correct diagnosis and appropriate management.

## 2. Etiology

**Figure 1:**
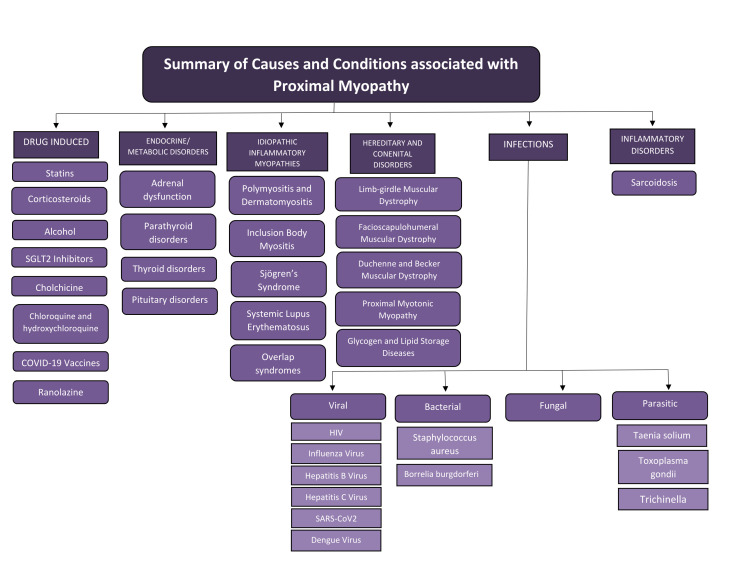
Flowchart presenting summary of all the causes and conditions associated with proximal myopathy

Multiple factors, such as toxins, infective agents, malignancies and endocrine disorders, contribute to the development of proximal myopathy^1^. It may also be observed in various idiopathic inflammatory myopathies (IIM) and hereditary/congenital myopathies. All the causes and conditions associated with proximal myopathy have been summarized in the form of a flowchart in Figure 1 and in more depth in Table 1.

**Table 1:** A summary of causes and conditions associated with Proximal Myopathy along with important features

Etiology	Mechanism leading to Myopathy
TOXINS	
Statins	· Decline in resting sarcolemmal chloride conductance (gCl) and chloride channel expression (ClC-1) · Variation in SLCO1B1 gene that encodes organic anion transporting protein (OATP1B1) · Reduced Coenzyme Q (CoQ) · In statin-induced necrotizing autoimmune myopathy, autoantibodies are produced against 3-hydroxy-3-methylglutaryl-CoA reductase which result in severe muscular manifestations
Corticosteroids	· Long term use of steroids leading to change in metabolism of skeletal muscle protein by transcriptional and growth factor (myostatine, IGF-2) modification
Alcohol	· Disturbance of balance between protein synthesis and degradation in skeletal and cardiac muscles · Ethanol has direct and indirect effects on mammalian target of rapamycin, mTOR · Long-term alcohol consumption decreases the translational efficiency of cellular machinery · Ethanol induces lipid peroxidation and depletion of serum antioxidants resulting in increased apoptosis of striated muscles
SGLT2 Inhibitors	· Improved mitochondrial activity, lowered protein turnover and inflammation, and higher energy intake as a result of chronic glycosuria are proposed as the cause of this drug-induced myopathy
Colchicine	· A genetic mutation in ABCB1 Gene (encoding for P-glycoprotein) may be a possible contributing factor in the colchicine myopathy
Chloroquine and hydroxychloroquine	· These drugs interfere with signaling pathways and transcriptional activity, interfere with lysosomal activity and autophagy, affect membrane stability, which can decrease the generation of cytokines and modify the levels of certain co-stimulatory molecules
COVID-19 Vaccine	· Inflammatory myositis has a temporal link to vaccination. · The antigenic target for COVID-19 vaccines, which are crucial for eliciting certain immune responses, has evolved to include the spike protein, the receptor-binding domain, and other structural proteins.
Ranolazine	· Drug interactions causing rhabdomyolysis are a possible cause of myopathy
ENDOCRINE AND METABOLIC DISORDERS	
Adrenal Dysfunction	· Adrenal overproduction leads to decreased plasma myoglobin and serum creatinine kinase, and consequently low myoelectric activity of skeletal muscles. · Adrenal underproduction leads to hyperkalemic and hyponatremic states which may cause proximal myopathy.
Parathyroid Disorders	· Parathyroid overactivity negatively affects the muscle metabolism leading to myopathy. · Hypocalcaemia in parathyroid underactivity may lead to myopathy in some cases.
Thyroid Disorders	· In thyroid overactivity, an elevation of metabolic activity that results in increased catabolism in muscle cells, may lead to myopathy. · In thyroid underactivity, glucosaminoglycan deposition in type II and type I muscle fibers leads to impaired contractility of the actin-myosin unit, decreased myosin ATPase activity, and a slower ATP turnover, all of which may lead to myopathy.
Pituitary Disorders	· Overactivity of pituitary gland as in acromegaly, may manifest with type II fiber atrophy. · Imbalance in production or secretion of pituitary hormones may lead to myopathy by influencing hormonal functions of other glands.
IDIOPATIC INFLAMMATORY MYOPATHIES	
Polymyositis and Dermatomyositis	· Infiltration of macrophages and activated CD8+ cytotoxic T cells into muscles can cause muscle inflammation and fatty infiltration, leading to myopathy
Inclusion Body Myositis	· Strong association with HLA variations (HLADRB103: 01 and HLA-B08: 01) · Muscle invasion by large granular lymphocytes (LGL) can be observed in all the cases of IBM, suggestive of a possible mechanism for myopathy
Sjögren’s Syndrome	· Common myopathies in patients of Sjögren’s Syndrome are myositis and necrotizing autoimmune myopathy · In a rare case of Sjögren’s Syndrome myopathy, the muscles were hyalinized but there were no inflammatory cells, and the symptoms did not improve after immunosuppressive therapy
Systemic Lupus Erythematosus	· Dermatomyositis, necrotizing myositis, and Acute necrotizing myopathy have been found to be associated with Systemic Lupus Erythematosus (SLE) · Macrophage activation syndrome (MAS) in this disease has been observed as an important cause of necrotizing myopathy in children
Overlap syndromes	· Anti-Ku antibodies are produced in the Scleroderma-Polymyositis Overlap Syndrome, which result in inflammatory myopathies. · Hypothyroid myopathy along with Anti-Ku antibodies overlap syndrome · Inflammatory myopathies overlap with myasthenia gravis also results in proximal muscle weakness
HEREDITARY OR CONGENITAL MYOPATHIES	
Limb-girdle Muscular Dystrophy	· Multiple individual genetic mutations that primarily cause protein deficiency or misfolding, lead to calpainopathy, dysferlinopathy, sacroglycanopathy, or anoctaminopathy depending on the gene involved in the mutation.
Facioscapulohumeral Muscular Dystrophy	· Somatic mosaicism, p13E-11 deletion, 4q-10q translocations, and other non-canonical alterations, molecular combining can lead to myopathy by displaying allelic combinations seen in FSHD.
Duchenne Muscular Dystrophy	· In DMD, premature protein translation truncation, results in non-functional and unstable dystrophin, leading to myopathy. It is brought about by nonsense mutations or frameshifting genetic mutations.
Becker Muscular Dystrophy	· In BMD, partly functional dystrophins with fewer spectrin-like repeats are formed with both F-actin and extracellular matrix binding domain. These unusual proteins formed lead to myopathy.
Proximal Myotonic Myopathy	· Alterations in RNA binding proteins and the disruption in gene due to untranslated repeat expansions in RNA might induce the multisystemic properties shared by both types of myotonic dystrophies.
Glycogen and Lipid Storage Diseases	· PYGM gene (11q13) mutations forms inactive enzymes which may induce myopathy. · Lipid Deposition Myopathy occurs when abnormal metabolism causes lipids to accumulate in skeletal muscle fibres, leading to lesions and degeneration.
INFECTIONS	
HIV	· HIV-associated myopathy can arise during any time of the infection. · Myopathy brought on by antiretrovirals, particularly nucleoside reverse transcriptase inhibitors (NRTI), is caused by mitochondrial damage. Zidovudine is the drug that results in the most offence.
COVID-19	· Critical illness myopathy (CIM) is caused by the inflammatory cytokine storm, coagulopathy, and macrophage activation that occur during prolonged critical care stay in hospitals
· Influenza Virus · Hepatitis Virus B (HBV) · Hepatitis Virus C (HCV) · Severe Acute Respiratory Syndrome Coronavirus 2 (SARS-CoV2) · Staphylococcus aureus · Dengue Virus · Borrelia burgdorferi · Fungal Myositis · Myositis by Trichinella · Myositis by Taenia solium · Myositis by Toxoplasma gondii	· Without infecting the muscle, myositis can develop as a result of pathogens stimulating the immune system. · The given entities may also cause infectious myositis targeting skeletal muscles. · Dengue virus has recently been reported as a cause of immune mediated necrotizing myopathies (IMNM)
INFLAMMATORY DISORDERS	
Sarcoidosis	· Cause unclear

### 2.1. Drug-induced Myopathies

As the current literature suggests, myopathy is an important and frequent complication of long-term use of numerous drugs which include statins, corticosteroids, alcohol, and SGLT-2 inhibitors. Antimalarials such as chloroquine and hydroxychloroquine have also been known to cause muscle wasting. Additionally, colchicine-induced myopathy, though uncommon, has also been reported. The nervous and muscular adverse effects (NMAE) associated with COVID-19 vaccination prompt further discussion regarding the mechanism and management of myopathy post-COVID-19 vaccination. Each of these drugs, along with myopathy as a side effect of ranolazine therapy, has been discussed with detail in the subsequent subsections.

#### 2.1.1 Statins

Myopathy is a well-known side effect of long-term statin use. Statin-associated myopathy symptoms (SAMS) include proximal muscle pain, weakness, tenderness, cramps, and fatigue^6,7^. SAMS are frequently observed in clinical practice, with an incidence ranging from 10% to 29%^8^. The risk of statin-induced myopathy is associated with a number of variables including patient characteristics (age, demographics, co-morbidities, genetics), pharmacological characteristics (particular statin molecule, dose, and pharmacokinetics), and drug interactions^9^. Individuals around the age of 65 years are more likely to develop myopathy and rhabdomyolysis as compared to younger population^10^. The risk for developing symptoms is also higher for those of Chinese or Japanese origin as Asians taking same doses of rosuvastatin have been reported to have plasma levels of the drug that are two times greater than the plasma levels observed in Caucasians^9^. In addition to this, statin-induced myopathy appears to develop more in people with chronic illnesses such diabetes, kidney disease, and cardiovascular disease^11^.

Myalgias can be multifactorial and are typically not accompanied by a feeling of weakness or an increased creatine kinase (CK) level^12^. The decrease in resting sarcolemmal chloride conductance (gCl) and expression of chloride channel (ClC-1) relates to the statin-induced muscle damage^8^. The genetic alteration of genes coding for statin metabolism adds to the possibility of myotonic damage i.e., genetic variation of SLCO1B1 gene which encodes for organic anion transporting protein (OATP1B1), responsible for entry of statin into the hepatocytes, is associated with an increased risk of myopathy^13^. Moreover, reduction in Coenzyme Q (CoQ) has been observed in patients with statin-induced myopathy as CoQ supplementation improves these muscular symptoms^14^. Most of the drug interactions of statins involving cytochrome P450 3A4, organic-anion-transporting polypeptides 1B1 (OATP1B1), and P-glycoproteins also induce muscle toxicity^15^. In stat^16^in-induced necrotizing autoimmune myopathy (SINAM), autoantibodies are produced against 3-hydroxy-3-methylglutaryl-CoA reductase (HMGCR) which result in severe muscular manifestations^17^. The majority of patients diagnosed with SINAM have been reported to be

taking atorvastatin, simvastatin, or rosuvastatin^18^. Moreover, rare cases of esophageal and pharyngeal muscle involvement that led to dysphagia and pneumonia respectively in patients with Anti-HMGCR antibodies have also been presented^19,20^.

Management of statin-induced myopathy includes switching to low-dose statins (including alternate day dosing) and other LDL-lowering drugs, such as bile acid-binding resins, ezetimibe, nicotinic acid, and PCSK9 inhibitors^21^. With a strong safety record to date, PCSK9 inhibitors significantly cut LDL-C by around 50 to 70%, without the need for concurrent statin therapy^22^. Recent studies have also shown more therapeutic alternatives such as bempedoic acid^22^.

#### 2.1.2 Corticosteroids

The long-term therapeutic use of steroids can also lead to the development of either chronic or acute myopathy^23^. While there have been case reports of steroid induced myopathy caused by inhaled corticosteroids and epidural, intramuscular, or intra-articular injections, the condition is usually caused by the use of oral and intravenous formulations^24^. When administered for four weeks or longer, doses greater than 10 mg prednisone equivalents/day cause the symptoms of muscle weakness to manifest^25^. However, acute steroid induced myopathy, in patients admitted to intensive care units, is associated with doses greater than 60 mg/day for 5 to 7 days^25^. Chronic steroid induced myopathy usually affects proximal muscles and shows Cushingoid appearance^23^. Multiple factors contribute to steroid induced myopathy, one of which is the change in metabolism of skeletal muscle protein by transcriptional and growth factor (myostatine, Insulin-like growth factor 2 (IGF-2)) modification^26,27^. Glucocorticoids induced myopathy is an important clinical feature among patients with acquired Cushing’s syndrome^24,28^.

#### 2.1.3 Alcohol

Among non-prescribed toxins, alcohol is the leading cause of myopathy^1^. This toxin impairs the balance between protein synthesis and degradation in skeletal and cardiac muscles^29^. Ethanol has direct and indirect effects on mammalian target of rapamycin mTOR, a protein that regulates the muscle mass^30^. Unlike steroids, long-term alcohol consumption decreases the translational efficiency of cellular machinery^31^. Ethanol-induced lipid peroxidation and depletion of serum antioxidants result in increased apoptosis of striated muscles and decreased muscle mass and strength^32^. Dystrophin, a membrane associated protein, is usually damaged by the free radicals thus leading to necrotizing muscle fibers^33^.

#### 2.1.4 SGLT2 Inhibitors

Sodium-glucose co-transporter 2 (SGLT2) inhibitor is a class of antidiabetic drugs that inhibits glucose absorption from the proximal tubule of the kidney and causes glycosuria^34^.SGLT2 inhibitors such as empagliflozin and dapagliflozin are reported to cause marked weight loss and muscle wasting in patients with diabetes mellitus^35^. Case reports suggest that SGLT2 inhibitors therapy results in muscle weakening, pain, and exercise intolerance in patients with diabetes^16^. Improved mitochondrial activity, low protein turnover and inflammation, and higher energy intake as a result of chronic glycosuria are some of the factors that have been hypothesized as the causes of myopathy induced by SGLT2 inhibitors^16^. Improvement in muscle symptoms has been reported after discontinuation of the medicine^16^. Recently, Anti-HMGCR/ immune-mediated necrotizing myopathy has also been reported in a patient following dapagliflozin administration with metformin^36^.

#### 2.1.5 Colchicine

Colchicine is an alkaloid with broad anti-inflammatory effects that is clinically used in gout and cardiovascular diseases^37^. Colchicine-induced myopathy is an uncommon side effect of colchicine therapy that manifests as an autophagic, vacuolar myopathy with painless proximal weakness of muscles^38^. This type of myopathy is more common in patients with digestive discomfort, bone marrow suppression, and liver and renal dysfunction^39^. Colchicine with statin co-administration (usually in treatment of gouty arthritis) causes rhabdomyolysis and neuromyopathy due to drug interactions, as both are metabolized by cytochrome P450 3A4^40^. P-glycoprotein has an important role in metabolism of colchicine^40^. It has been reported in a case series that ABCB1 (gene coding for P-glycoprotein) genetic variation can be a probable contributing cause of the colchicine myopathy^41^.

#### 2.1.6 Chloroquine and hydroxychloroquine

Chloroquine and hydroxychloroquine are the conventional anti-malarial drugs that are also used in the treatment of rheumatoid arthritis and other inflammatory rheumatic diseases^42^. Chloroquine (CQ) and hydroxychloroquine (HCQ) myopathy affects the proximal musculature of limbs and is associated with cardiomyopathy with significant dysphagia and respiratory failure^43^. These drugs interfere with signaling pathways and transcriptional activity, interfere with lysosomal activity and the process of autophagy, affect membrane stability, which can decrease the generation of cytokines and modify the levels of certain co-stimulatory molecules^42^. CQ and HCQ induced cardiotoxicity shows nonspecific pathological findings and is overlapped with vacuolated myopathy^44^.

#### 2.1.7 Covid-19 Vaccine

Nervous and muscular adverse effects (NMAEs) post immunization against COVID-19 have received a lot of attention^45^. The antigenic target for COVID-19 vaccines, which are crucial for eliciting certain immune responses, has evolved to include the spike protein, the receptor-binding domain, and other structural proteins^46^. A systematic review and meta-analysis conducted by Jiaxin Chen et al.,^47^ showed that NMAEs associated with COVID-19 vaccine, particularly headache and myalgia, were frequent, albeit the severe and life-threatening ones were uncommon. The incidence of NMAEs was 29.2% in the vaccinated group and 21.6% in the control group in a total of 15 randomized, blinded, controlled clinical trials (phase1/2)^47^. From this we can infer that the incidence of NMAEs was 8% higher in individuals who had received COVID-19 vaccine. However, as the follow-up period of clinical trials was short, and phase 3 trials were still running, the incidence rates may have varied^47^. Additionally, a case involving an 81-year-old patient who developed myositis and arm cellulitis post COVID-19 vaccine has recently been described^48^. A rare incidence of a major adverse event following COVID-19 mRNA immunization gives an illustration of how inflammatory myositis exhibits a temporal link to vaccination^49^. Moreover, according to the results from mRNA vaccine clinical trials, 7 out of 37,000 vaccinated participants developed Bell’s palsy^50^.

#### 2.1.8 Ranolazine

The anti-ischemic drug Ranolazine reportedly causes proximal myopathy in patients with long-term statin therapy^51^. Rhabdomyolysis due to drug interaction is a suggestive cause of myopathy^52^. However, a case report of ranolazine-induced myopathy has reported an increase in serum CK in a patient with no statin history^53^. Additionally, lipid-storage myopathy in proximal muscles has also been reported to be associated with ranolazine therapy^54^.

### 2.2. Endocrine and Metabolic Disorders

Several endocrine and metabolic disorders have been known to cause muscle wasting. The mechanism of myopathy observed in adrenal dysfunction including Cushing syndrome and Addison’s disease, in parathyroid disorders including hyperparathyroidism and hypoparathyroidism, in thyroid disorders including hyperthyroidism and hypothyroidism, and in pituitary dysfunction has been summarized under separate headings in the following subsections.

#### 2.2.1 Adrenal Dysfunction

Both adrenocortical overproduction and adrenal insufficiency cause myopathy^55^. In patients with Cushing syndrome, a decrease in plasma myoglobin and serum CK has been observed. There is also a decrease in myoelectric characteristics of all the fatigued muscles^28,56^. In Addison’s disease, inflammatory myopathy with muscle tenderness is commonly observed^57^. Hyperkalemic neuromyopathy has also been reported previously in many cases^58^. Moreover, severe hyponatremia also renders weakening effects on muscles as observed in Schmidt’s syndrome^59^.

#### 2.2.2 Parathyroid Disorders

Hyperparathyroidism has been linked to muscle dysfunction and is associated with muscular weakness, myopathy, and poor postural stability, as an excess of parathyroid hormone (PTH) appears to have negative effects on skeletal muscle metabolism^60^. Type II atrophy is visible on muscle biopsy with no fiber necrosis, regeneration, or inflammatory infiltrate^61,62^. Hypoparathyroidism rarely shows any muscle involvement, but the associated hypocalcemia causes muscle tetany (increased neuromuscular excitability) and mild weakness^63^.

#### 2.2.3 Thyroid Disorders

**Figure 2:**
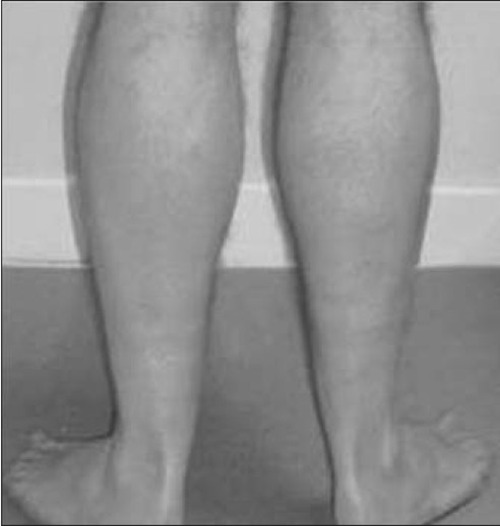
Calf Muscle Hypertrophy observed in Hyopthyroid myopathy: Hoffman Syndrome Courtesy of: Sundarachari et al.,^70^ under the conditions of Creative Commons Attribution-NonCommercial-ShareAlike 4.0 License, no changes were made to the figure and/or figure legend.

**Figure 3:**
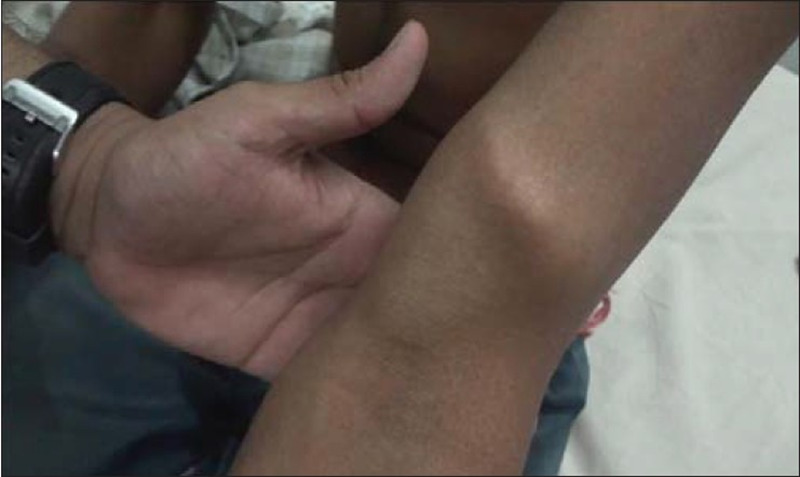
Percussion Myotonia observed in Hyopthyroid myopathy: Hoffman Syndrome Courtesy of: Sundarachari et al.,^70^ under the conditions of Creative Commons Attribution-NonCommercial-ShareAlike 4.0 License, no changes were made to the figure and/or figure legend.

About 30 to 80 percent patients of hypothyroidism experience neuromuscular symptoms^63^. In hypothyroid myopathy, glucosaminoglycan deposition in type I and type II muscle fibers leads to impaired contractility of the actin-myosin unit, decreased myosin ATPase activity, and a slower ATP turnover; all of which contribute significantly to the skeletal muscle damage^64,65^. Rare types of hypothyroid myopathies with pseudohypertrophy of muscles are also found which include the Hoffmann’s syndrome in adults (Figure 2 and Figure 3), and Kocher-Debre-Semelaigne syndrome in children^66,67^. Hyperthyroid myopathies are usually observed in thyrotoxic patients^68^. The etiology of muscle failure in hyperthyroidism is most likely to be an elevation of metabolic activity that results in increased catabolism in muscle cells. Most Asian patients present with sudden onset of hypokalemia with muscle paralysis, referred to as thyrotoxic periodic paralysis (TPP); one of the complications of hyperthyroidism^69,70^.

#### 2.2.4 Pituitary Disorders

Pituitary gland disorders rarely contribute directly to any type of myopathy^71^. However, cases of myopathy in acromegaly (due to overproduction of growth hormone) have been reported with type II fiber atrophy^72^. Although these muscles frequently appear larger, they often generate less force. Instead of blood growth hormone levels, myopathy severity is correlated with the time since onset of acromegaly^73^. As the pituitary hormones significantly influence endocrine functions of other glands, any imbalance in their production or secretion can indirectly lead to myopathy

### 2.3 Idiopathic Inflammatory Myopathies

Inflammatory myopathies including polymyositis, dermatomyositis, and Inclusion Body Myositis have been reported to be associated with symptoms of muscle weakness and muscle wasting. In addition to these, myopathy is also observed in Sjogren Syndrome, Systemic Lupus Erythematosus, and Overlap syndromes. These conditions have been thoroughly explored in the subheadings below.

#### 2.3.1 Polymyositis and Dermatomyositis

The inflammatory myopathies are a diverse collection of acquired skeletal muscle diseases that can be acute, subacute or chronic. Polymyositis (PM) and dermatomyositis (DM) exhibit both moderate to severe muscle weakness as well as chronic muscle inflammation^74^. As seen on histopathology, the muscle infiltrate is composed of macrophages and activated CD8+ cytotoxic T cells, which occasionally invade non-necrotic muscle fibers that express MHC-I proteins (Major histocompatibility complex class I)^75^. In idiopathic inflammatory myopathy (IIM) patients, magnetic resonance imaging (MRI) shows chronic disease pattern (fatty infiltration) and muscle inflammation^76^. The knowledge of polymyositis has evolved substantially since the Bohan and Peter et al.,^77^ criteria were published in 1975. The newly published European League Against Rheumatism (EULAR)/American College of Rheumatology (ACR) adult PM/DM and juvenile DM response criteria, as well as the myositis disease activity core measurements supported by International Myositis Assessment & Clinical Studies (IMACS) have now been proposed^78^. A detail of the new EULAR/ACR IIM classification criteria will be out of scope of this narrative review, however, the developments made in this regard can be read in Leclair and Lundberg’s review^78^. Due to its rarity, polymyositis can only be differentiated when other disease groups have been ruled out. While making differential diagnoses, the possibility of connective tissue disease overlap myositis and immune mediated necrotizing myopathy should be taken into consideration^79^.

#### 2.3.2 Inclusion Body Myositis

Inclusion body myositis (IBM) is the most prevalent acquired myopathy above the age of 50 with about 20% patients developing symptoms in their forties. The frequency of IBM ranges from 24.8 to 45.6/1,000,000^80^. Classically long finger flexor and quadriceps weakness with a slightly increased CK level are important clinical presentations in IBM. Additionally, treatment resistance to immunosuppressants should spur investigations^81^. Genetic studies show a substantial hereditary relationship with HLA variations (HLADRB103: 01 and HLA-B08: 01) that are a component of widespread autoimmune diseases and 8.1 ancestral MHC haplotype^82^. Muscle invasion by large granular lymphocytes (LGL) was observed in 100% of IBM cases as compared to 3.5% DM/PM cases. It was also reported that 60% of IBM patients had enlarged CD8+ CD57+ T LGL cells. This neoplastic proliferation of T-cells results in immunotherapy resistance^83,84^. Only a small number of IBM subgroups, such as those with rapid onset, with creatine phosphokinase (CPK) levels above 15 U/N, or those associated with other autoimmune diseases, have demonstrated benefit from immunosuppressive therapy^85^. Myo-degenerative pathways have been preferred targets for therapy in majority of studies in case of failure of immunosuppressants to yield desirable results^86^.

#### *2.3.3 Sjögre*n’s Syndrome

Individuals with primary Sjögren’s Syndrome (SS) typically experience muscle soreness and/or muscular weakness. Myositis is seen in less than 3% of patients^87^. In primary SS, as skeletal involvement is relatively infrequent, assessment for the likelihood of mixed connective tissue disease should be considered as well^87^. Myositis and necrotizing autoimmune myopathy are common myopathies in patients with SS^88^. A rare case of myopathy in SS has been reported to be majorly affecting hyalinized muscles, however, no improvement of symptoms was observed after immunosuppressive therapy^89^.

#### 2.3.4 Systemic Lupus Erythematosus

Involvement of the musculoskeletal system occurs in about 4% to 16% of Systemic Lupus Erythematosus (SLE) patients^90^. The weakening, myalgia, and atrophy are frequent symptoms of proximal muscles of upper and lower extremities, although severe weakness is unusual^90^. Dermatomyositis (38%), and necrotizing myositis (50%) are the two histological categories that are most commonly associated with SLE^91^. Acute necrotizing myopathy without myositis, though rare, has also been reported^92^. Recently, Macrophage activation syndrome (MAS) in SLE has been observed as an important cause of necrotizing myopathy in children^93^.

#### 2.3.5 Overlap syndromes

Patients with overlap syndromes (inflammatory rheumatic disorders), present clinical signs that may be related to several different immunological diseases^94^. Proximal myopathy has been observed in many overlap syndromes. More recently, a patient with SLE and Sjögren's overlap syndrome, was diagnosed with sporadic late-onset nemaline myopathy (SLONM)^95^. In Scleroderma-polymyositis overlap syndrome, anti-Ku antibodies are produced which cause inflammatory myopathies. Recently, a case of hypothyroid myopathy along with anti-Ku antibodies overlap syndrome has also been reported^96^. A rare syndrome, in which inflammatory myopathies overlap with myasthenia gravis, causing progressive proximal muscle weakness and weight loss in patient has also been reported in an African-American patient^97^.

### 2.4. Hereditary or Congenital Myopathies

In discussion pertaining to causes and conditions associated with proximal myopathy, symptoms of muscle weakness associated with hereditary disorders such as limb girdle muscular dystrophies (LGMDs), facioscapulohumeral muscular dystrophy (FSHD), Duchenne and Becker Muscular Dystrophy, and myotonic muscular dystrophies should be equally emphasized on. In addition to these, we also discuss myopathy observed in various glycogen and lipid storage diseases.

#### 2.4.1 Limb-girdle Muscular Dystrophy

The limb girdle muscular dystrophies (LGMDs) are skeletal muscle-specific hereditary disorders with significant proximal muscle weakness^98^. A thorough physical examination and a detailed study of the pattern of muscle weakening may prove helpful in identification of a specific kind of LGMD. There are at least 30 genetic etiologies associated with LGMD, with this number continually increasing. Francesca Magri et al.^99^ found an 84:16 percent distribution for the recessive variants, making recessive variants more prevalent than the dominant ones. Individual genetic mutations that primarily cause protein deficiency or misfolding give rise to the LGMD subtypes. The mutation in proteins results in glycosylation modification, mitochondrial malfunction, and mechanical transduction^100^. About 30% of LGMD patients are thought to have calpainopathy (mutation in the CAPN3 gene), which has been documented more frequently than other most widespread subtypes^101^. Other subtypes of LGMD may exhibit dysferlinopathy (mutations in the DYSF gene), sarcoglycanopathy (missense mutation within the *SGCA, SGCB, SGCG,* and SGCD genes), and anoctaminopathy (mutation in ANO5 gene) that can have dystrophic effects on the muscle fibers^102^. Investigations in the pre-clinical models, including the mouse model employing AAV (Adeno-Associated Virus)-mediated gene replacement therapy, show therapeutic effectiveness and give justification for further research in clinical studies^103^.

#### 2.4.2 Facioscapulohumeral Muscular Dystrophy

As suggested by its name, facioscapulohumeral muscular dystrophy (FSHD) entails weakening of the muscles supporting the scapula, and humerus along with facial muscles. FSHD demonstrates an autosomal dominant pattern of inheritance^104^. The two types (type 1 and type 2) are reported with 19:1 prevalence rate^104^. The condition of patients with early-onset FSHD worsens rather quickly. They tend to have more severe muscular weakness, and manifest systemic symptoms more frequently than those with adult-onset FSHD^105^. The double homeobox protein 4 (DUX4) gene expresses abnormally in skeletal muscle, independent of the complex and varied hereditary causes of FSHD, causing strong cytotoxicity by influencing cellular death, oxidative stress, and muscle growth pathways^106^. Using the blot hybridization probe p13E-1, Southern blotting is a standard method for FSHD diagnosis. Methylation study of the D4Z4 area and SMCHD1 sequencing on chromosome 18 are carried out for the diagnosis of FSHD type 2. By finding somatic mosaicism, p13E-11 deletion, 4q-10q translocations, and other non-canonical alterations, molecular combining can directly display allelic combinations associated with FSHD^107^. Losmapimod, a p38MAPK inhibitor that has been demonstrated to lower DUX4 levels, is now a subject of the first study addressing pathogenic mechanism underlying FSHD. Newer options for treatment are provided by developments in the molecular mechanisms of DUX4 toxicity and targeting of gene therapy to the FSHD locus^108^.

#### 2.4.3 Duchenne and Becker Muscular Dystrophy

The dystrophinopathies are a collection of X-linked recessive disorders brought on by Duchenne muscular dystrophy (DMD) gene mutations^109^. DMD, the most prevalent and severe phenotype, affects 1 in 5000 male live births. The severity spectrum of the Becker muscular dystrophy (BMD), however, is substantially larger^110,111^. In DMD patients, deletions make up around 60 to 70 percent, duplications make up 5 to 15 percent and collectively point mutations, minor deletions, or insertions make up 20 percent of all the mutations that have been reported to occur^112^. In contrast, 60 to 70 percent of mutations in patients with BMD are deletions, 20 percent are duplications, and 5–10 percent are point mutations, minor deletions, or insertions^112-114^. In DMD, premature protein translation truncation, brought about by nonsense mutations or frameshifting mutations, results in non-functional and unstable dystrophin. However, In BMD, changes in the midsection of gene preserve the reading frame and enable synthesis of dystrophins with fewer spectrin-like repeats with both F-actin and extracellular matrix binding domains, making the proteins partly functional^115^. A clinical examination indicates muscular pseudohypertrophy in the calf muscles, however, more proximally situated muscles such as the quadriceps and other limb-girdle muscles may also exhibit atrophy (lower limbs are more likely to be affected than the upper limbs)^116^. *In vivo*, the ability of dystrophin to be restored to both its wild form and its shortened version highlights the ability of CRISPR technology to treat a variety of DMD-causing mutations and, potentially, its clinical use^117,118^.

#### 2.4.4 Proximal Myotonic Myopathy

The myotonic muscular dystrophies clinically present with progressive muscular weakness, myotonia, cardiac conduction disturbance, and cataracts. These are autosomal dominant disorders classified into: Muscular Dystrophy type 1 (MD1) and Muscular Dystrophy type 2 (MD2). MD2, also called Proximal Myotonic Myopathy (PROMM) causes weakness of proximal musculature and prominent pain. However, individuals with MD2 are more likely to have distal weakness of the dorsiflexors in the ankle, the long finger flexors, and the facial muscles^119^. In PROMM, the nucleic acid-binding protein (CNBP) gene on chromosome 3q21 exhibits an unstable tetranucleotide CCTG repeat expansion in intron 1^120^. In healthy people, the size of the (CCTG)n repeat is less than 30 repetitions, however patients with myotonic dystrophy type 2 have a wide range of expansion sizes. The smallest mutations that have been recorded range between 55 and 75 CCTG, while the greatest expansions have been estimated to reach 11,000 repetitions^120,121^. A persuasive explanation of how untranslated repeat expansions in RNA might induce the multisystemic properties shared by both types of myotonic dystrophy is provided by the alterations in RNA binding proteins and the disruption in gene splicing^122^. With the use of regular PCR, Southern blot analysis, and the PCR repeat-primed test, a CNBP CCTG expansion may be found more than 99 percent of the time^123^.

#### 2.4.5 Glycogen and Lipid Storage Diseases

A metabolic myopathy known as glycogen storage disease type V (McArdle disease) is characterized by an aversion to exercise that manifest as fast exhaustion, myalgia, and cramping in working muscles. Isometric activity or prolonged aerobic exercise typically causes symptoms to appear^124^. Patients with McArdle disease have PYGM gene (11q13) mutations, which render the enzyme inactive. The PYGM gene exons 1 and 17 contain the mutation hotspots, however half of the instances observed have nonsense mutations^125,126^. The term "lipid deposition myopathy" (LSM) refers to a class of hereditary metabolic myopathies in which abnormal metabolism causes lipids to accumulate in skeletal muscle fibers, leading to lesions and degeneration^127^. Although the progression of the disease might vary, the primary clinical signs are growing muscular weakening and movement intolerance^127^.

### 2.5 Infections

Symptoms of muscle weakness observed in various viral, bacterial, fungal, and parasitic infections have been discussed with detail in the subsequent sections.

#### 2.5.1 HIV

HIV-associated myopathy typically manifests as symmetrical proximal muscle weakness that worsens over the course of weeks to months. Skeletal muscle involvement may arise at any stage of HIV infection and is sometimes a patient's first sign of illness^128-130^. Myopathy caused by antiretrovirals, especially nucleoside reverse transcriptase inhibitors (NRTI), is hypothesized to be due to mitochondrial damage. The drug that causes the most offence is zidovudine^130^. Based on immunohistology for MHC class I antigen and histochemical reaction for cytochrome oxidase, a myopathy can be correctly identified as HIV myositis or zidovudine myopathy^129^. Myopathies are treated symptomatically in HIV patients, but if a definitive cause is found, then the underlying disease should be treated as necessary^131^.

#### 2.5.2 Covid-19

In the context of severe acute respiratory syndrome coronavirus 2 (SARS-CoV2), muscular involvement includes myalgia, myositis, as well as critical-illness myopathies^132^. An extra-pulmonary manifestation of COVID-19 is skeletal muscle myopathy which can range from mild myalgia to myositis or rhabdomyolysis, affecting up to one-third of symptomatic individuals^133^. Patients with COVID-19 who require lengthy ICU stays and mechanical ventilation may be at risk for developing severe short- and long-term effects of intensive care unit acquired weakness (ICUAW)^134^. During extended critical care, the inflammatory cytokine storm together with coagulopathy and macrophage activation cause skeletal muscle degeneration, resulting in critical illness myopathy (CIM)^135^.

#### 2.5.3 Other Infections

Myositis can occur as a result of stimulation of immune system by various pathogens, without actually infecting the muscle^136^. However, these microorganisms may also result in infectious myositis, which is skeletal muscle infection.These infectious agents include influenza viruses, hepatitis B virus (HBV), hepatitis C virus (HCV), Staphylococcus aureus and various fungi^137^. There have been several reports of benign acute myositis during influenza A and B outbreaks^138^. Acute pain and difficulties in walking often appear 3 days following the start of influenza symptoms^138^. The muscles are sensitive to palpation, occasionally exhibiting localized soft tissue edema, but seldom exhibiting overlying skin redness or pronounced muscular warmth^138^. The few documented muscle samples that have undergone histopathologic analysis reveal regions of muscle degeneration, sporadic sites of frank necrosis, and mild inflammatory infiltrates^139^. Dengue virus has recently been reported as a cause of immune mediated necrotizing myopathies (IMNM)^140^. Streptococcus is the most frequent cause of bacterial myositis that is not mediated by hematogenous dissemination, whereas other myositis are often caused by polymicrobial infection following trauma^141^. Recently, proximal muscle weakness in the legs caused by Borrelia burgdorferi was also reported^142^. According to recent reports, myositis can be caused by parasitic and fungal infections. The most frequent causes of parasite myositis are Trichinella, Taenia solium, and Toxoplasma gondii. Fungal myositis is uncommon and typically occurs in immunocompromised hosts^137,141^.

### 2.6 Miscellaneous Cause

Other miscellaneous causes associated with myopathy include:

#### 2.6.1 Sarcoidosis

An inflammatory disease affecting several systems, sarcoidosis has an unclear cause. The most frequently affected organ is the lung^143^. It is rare for the disease to develop in the musculoskeletal system^143^. However,about 0.5 to 2.5 percent of all sarcoidosis patients have muscular symptoms^144^. There are several types of muscular sarcoidosis described. Subacute or chronic muscular weakness and/or myalgia may originate from either nodular or diffuse muscle involvement^144,145^. Proximal muscle weakness and myalgia in individuals with a history of sarcoidosis should raise suspicion. Muscular involvement in this condition can only be verified through muscle imaging^146^. The conventional treatment for sarcoidosis is corticosteroid medication, which prevents the generation of cytokines including interlukin-2 (IL2), interferon (IFN), and tumor necrosis factor (TNF), causing granulomatous lesions to regress^147,148^. Recently, an unusual case of sarcoidosis with a history of polyarthritis, stomach discomfort, and proximal muscle weakness has been reported^149^.

## **3. **Conclusion

Multiple factors contribute to the pathophysiology of proximal myopathy. A number of events have been elucidated that lead to the development of muscle weakness. These include toxins and other infectious agents, and many endocrine and metabolic disorders. Idiopathic inflammatory myopathies, and hereditary and congenital disorders have been reported to be associated with this phenomenon as well. Inflammatory disorders including sarcoidosis exhibit similar manifestations of muscle weakness and myalgia. A detailed discussion on these has been provided in our review with the aim of exploring available literature. Although these events leading to proximal myopathy have been described discretely in previous literature, we provide an insight into the causes and conditions associated with this phenomenon in the form of a single compact and comprehensive review with new developments and recent evidence to facilitate the timely diagnosis and ultimately appropriate management of patients presenting with proximal myopathy.
